# Supplementary material for: Cockayne Syndrome Linked to Elevated R-Loops Induced by Stalled RNA Polymerase II during Transcription Elongation
Source: Nat Commun. 2024 Jul 17;15:6031. doi: 10.1038/s41467-024-50298-w (PMC11255242; doi:10.1038/s41467-024-50298-w)
Supplement: Supplementary file 1 — Supplementary Information [file 41467_2024_50298_MOESM1_ESM.pdf]

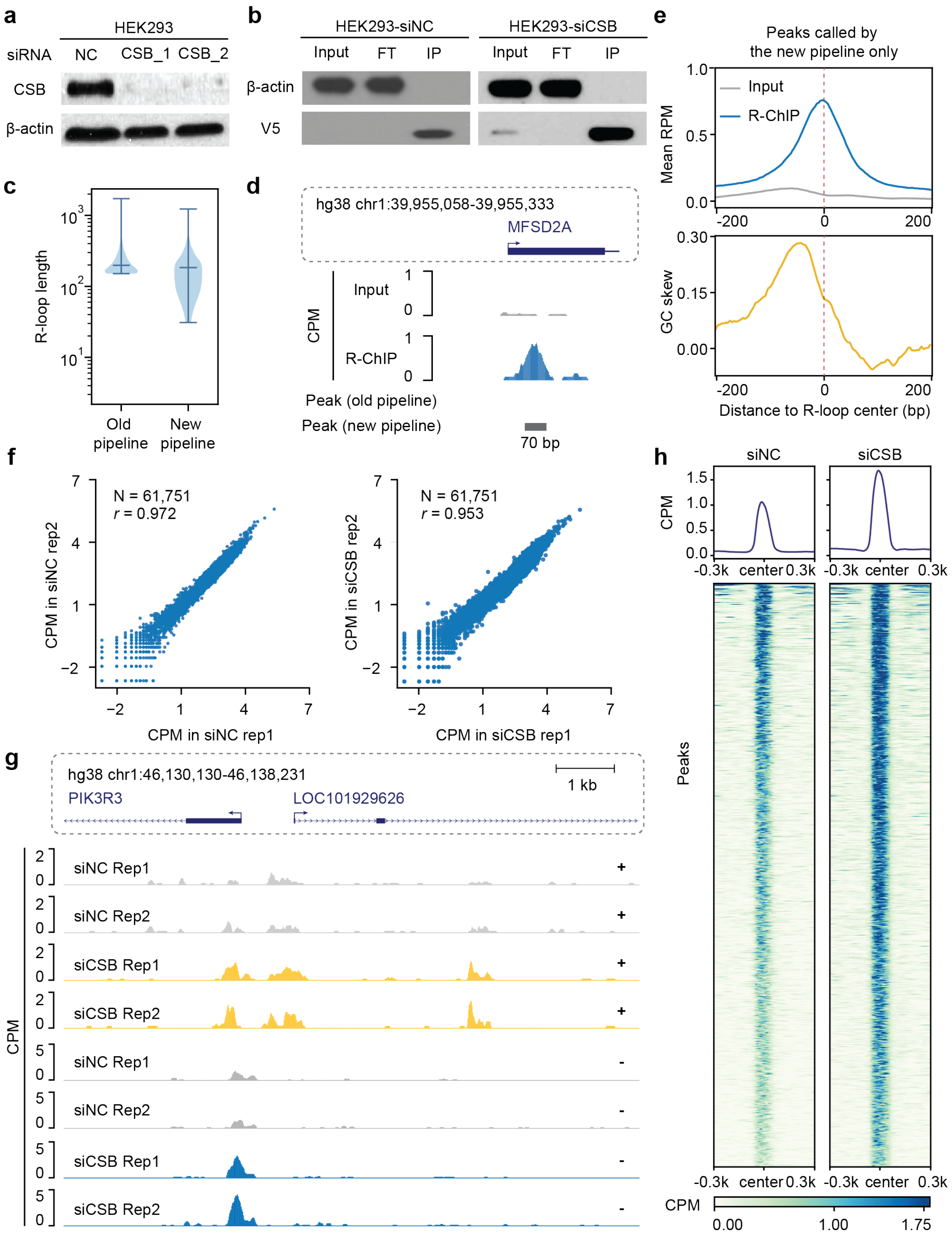
a
HEK293
siRNA NC CSB_1 CSB_2
CSB
β-actin
b
HEK293-siNC
HEK293-siCSB
Input FT IP
β-actin
V5
c
R-loop length
Old pipeline
New pipeline
d
hg38 chr1:39,955,058-39,955,333
MFSD2A
CPM
Input
R-ChIP
Peak (old pipeline)
Peak (new pipeline)
70 bp
e
Peaks called by the new pipeline only
Mean RPM
Input
R-ChIP
GC skew
Distance to R-loop center (bp)
f
N = 61,751
r = 0.972
CPM in siNC rep2
CPM in siNC rep1
N = 61,751
r = 0.953
CPM in siCSB rep2
CPM in siCSB rep1
g
hg38 chr1:46,130,130-46,138,231
1 kb
PIK3R3
LOC101929626
CPM
siNC Rep1 +
siNC Rep2 +
siCSB Rep1 +
siCSB Rep2 +
siNC Rep1 -
siNC Rep2 -
siCSB Rep1 -
siCSB Rep2 -
h
siNC
siCSB
CPM
-0.3k center 0.3k
Peaks
CPM
0.00 1.00 1.75

Supplementary Figure 1. Quality control and the modified R-loop peak calling pipeline.

(a) Knockdown efficiency in HEK293 cells with two independent siCSBs for 72 hours. NC: scrambled siRNA serving as a negative control. CSB was detected with a specific antibody and β-actin served as a loading control.

(b) Western blot validating the IP efficiency of V5 antibody used in R-ChIP. Input: fragmented chromatin before IP. FT: flow through chromatin after IP. IP: V5-enriched chromatin.

(c) R-loop length distribution comparing between the previous published pipeline and the newly modified pipeline.

(d) Genome browser tracks showing a region containing R-loop signals with a peak identified only according to the newly modified peak-calling pipeline. Gene annotations are shown on top.

(e) Metaplot (Blue) and GC-skew (Orange) profile of R-loops called by the newly modified peak-calling pipeline. Signals are centered on the R-loop summit around 0.2 kb surrounding regions.

(f) Correlative analysis of biological replicates of sequenced R-ChIP libraries.

(g) Genome browser tracks displaying two replicated signals in the identical region depicted in figure 1b.

(h) Heatmap of R-loop signals in siNC- and siCSB-treated cells.

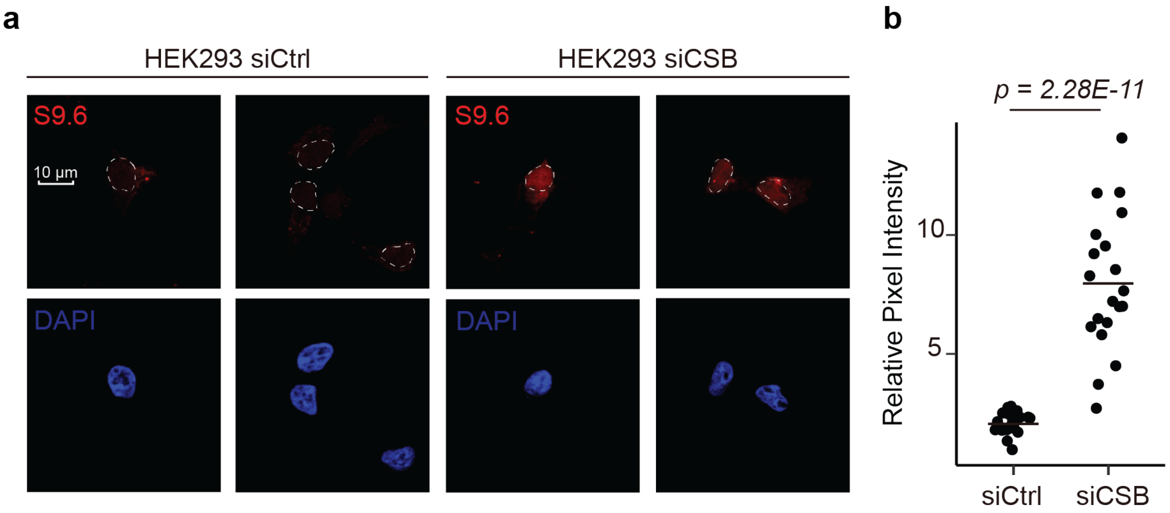

Supplementary Figure 2: S9.6 staining and quantification on CSB KD HEK293 cells.

(a) Immunofluorescence images illustrating S9.6 signals (R-loop marker, stained in red) and nuclei (stained in blue) in HEK293 cells treated with siCtrl or siCSB. Note that cytosolic S9.6 signals may encompass various RNA structures detected by the antibody.

(b) Quantitative analysis of nuclear S9.6-stained signals in siCtrl and siCSB-treated HEK293 cells. Relative pixel intensity was determined by subtracting background from individual pixel signals. Statistical analysis was performed using a two-tailed unpaired t-test (n=20 foci for both siCtrl and siCSB treated cells).

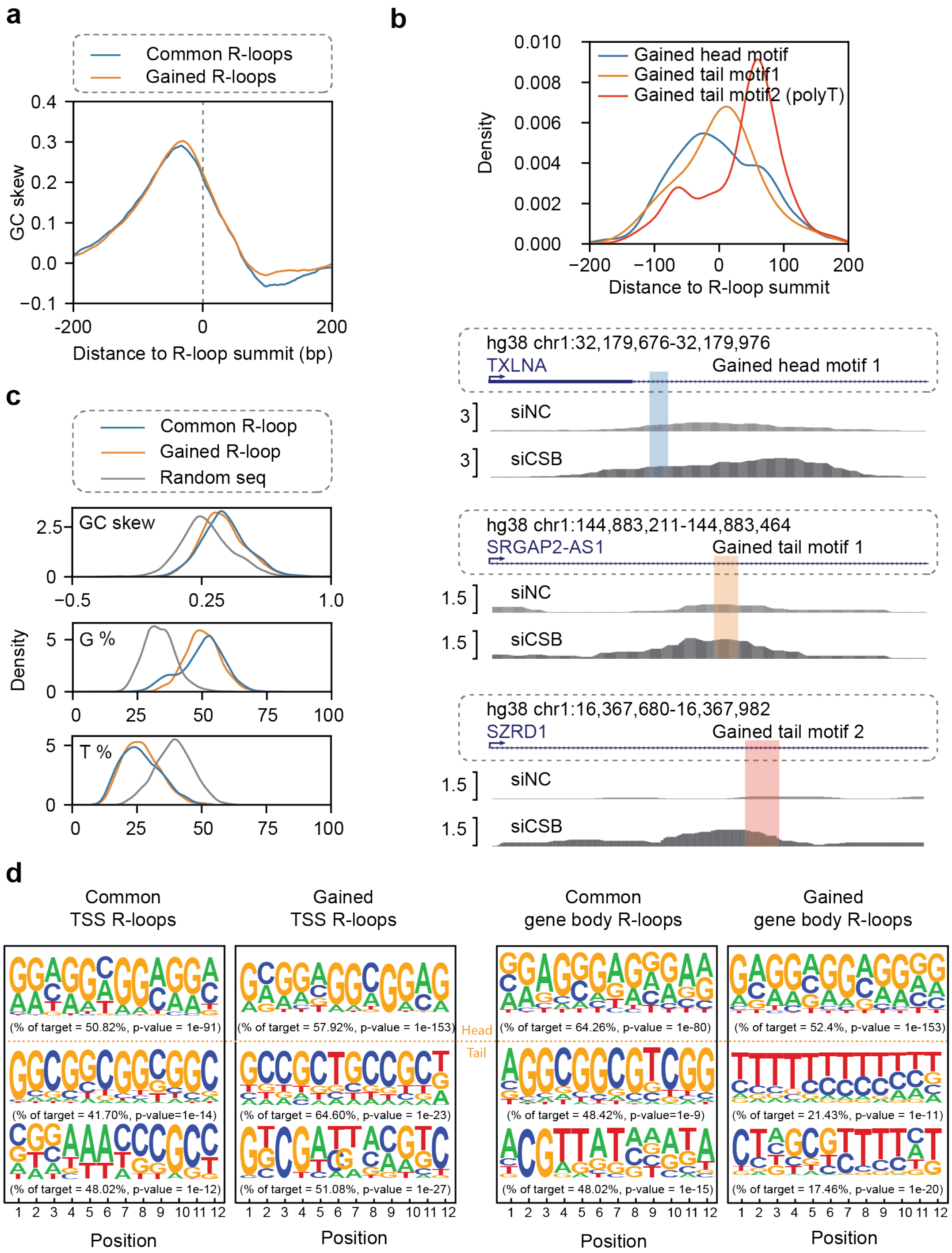

Supplementary Figure 3: R-loop features and enriched motifs.

(a) GC skew on R-loop signal-containing regions. The average of GC-skew (G - C/G + C) in common (blue) and gained (Organ) R-loop regions in siNC and siCSB-treated cells. GC-skew was calculated by using a window size of 50 bp and a step size of 1 bp.

(b) Three motifs enriched in Gained R-loops and their distributions relative to the summed R-loop peak (top) and genome browser tracks showing representative examples of three motifs enriched in gained R-loops(bottom). Gene annotations are shown on top of the tracks.

(c) GC skew, G percentage and T percentage distributions. Top: Maximum GC skew distribution of individual R-loops in common R-loops, gained R-loops and randomly selected genome sequences. Middle: Maximum G percentage distribution of individual R-loops in common R-loops, gained R-loops and randomly selected genome sequences. Bottom: Maximum T percentage distribution of individual R-loops in common R-loops, gained R-loops and randomly selected genome sequences. All values were calculated by using a window size of 50 bp and a step size of 1 bp.

(d) Motif analysis on TSS (Left) and genebody R-loops (Right). In each table, the top enriched motif in the head and two most enriched motifs in the tail are displayed as in the main Figure 1g. Enriched motifs among common (shared by siNC and siCSB) and gained (uniquely detected with siCSB) are separated displayed along with the frequency of each motif and associated p-value.

a

Distance to 3'SS	poly T R-loop %
< 10 bp	0
10 -100 bp	2.5
100 - 500 bp	10.5
500 - 1k bp	9.1
1k - 5k bp	26.7
> 5k bp	51.1

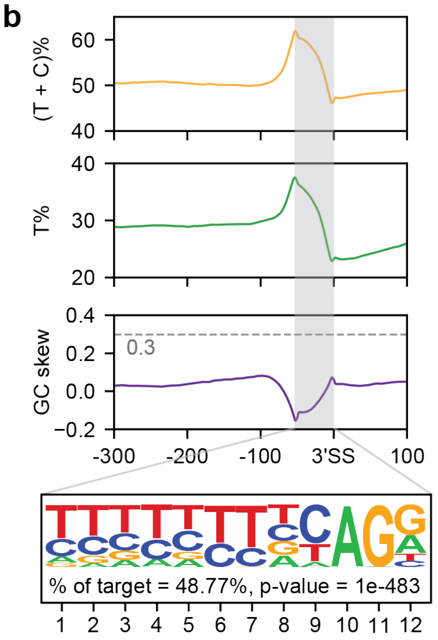

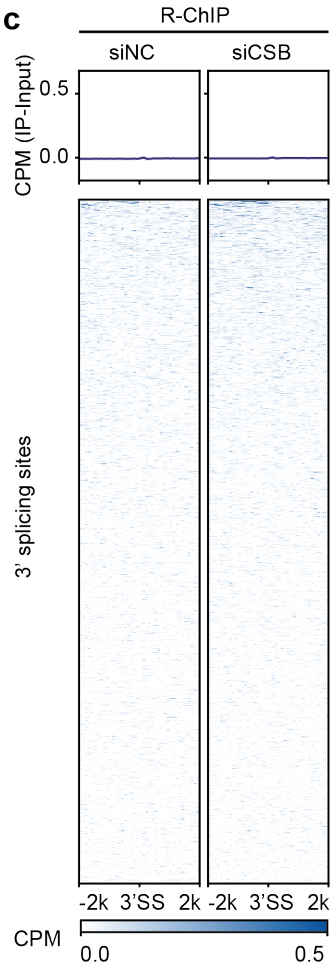

Supplementary Figure 4: T-runs profiles at 3' splice sites.

(a) Distance distribution of intronic T-run associated R-loop relative to their closest 3' splice sites.

(b) (T + C) percentage, T percentage and GC skew at 3' splice sites with associated motif.

(c) Lack of R-loop signals as indicated by the heatmap at 3' splice sites under siNC and siCSB treatment conditions.

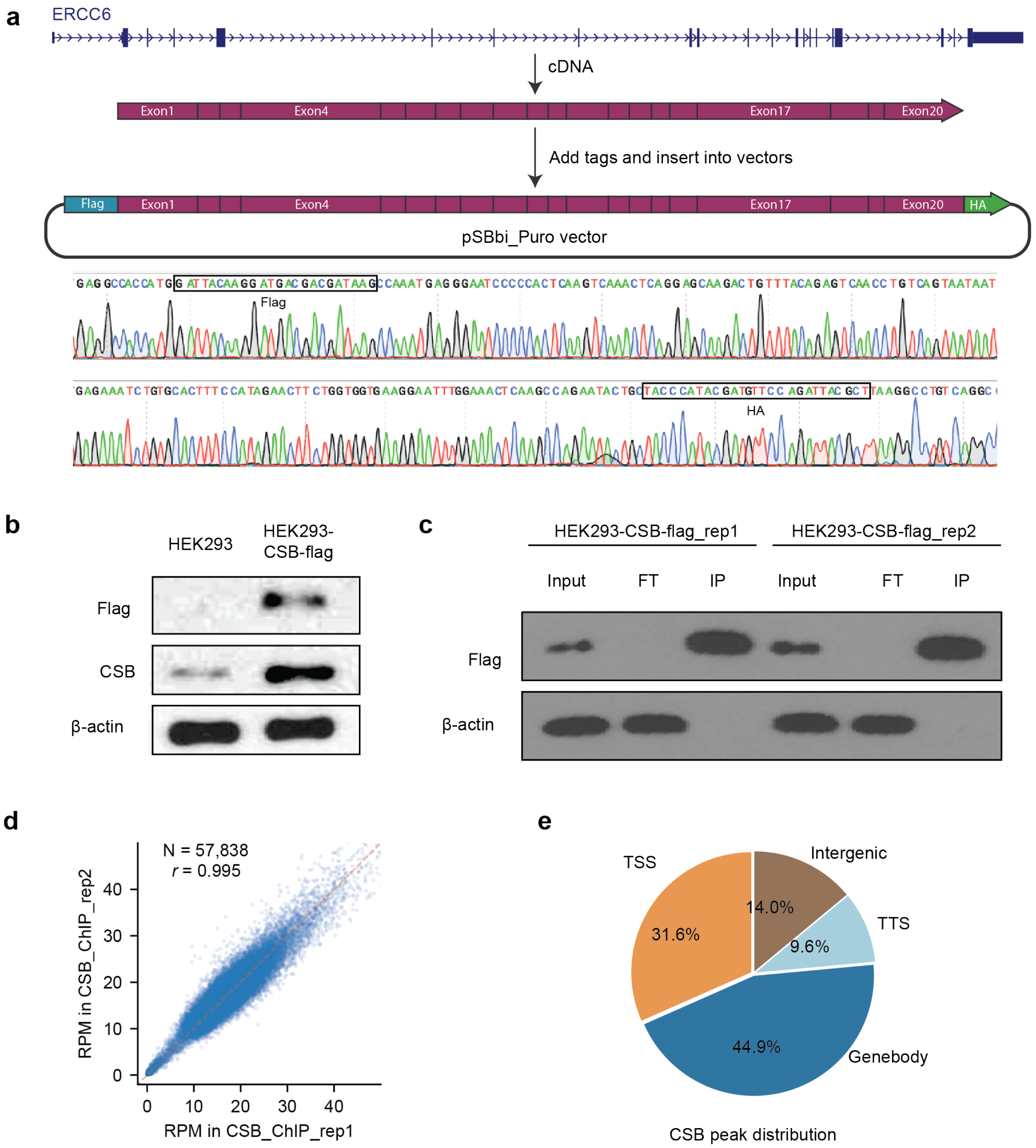

Supplementary Figure 5: Quality control and construct design for CSB ChIP-seq.

(a) Clone of CSB cDNA into the pSBbi_Puro vector for overexpression in HEK293 cells. CSB was N-terminally tagged with Flag and C-terminally tagged with HA and the clone was verified by Sanger sequencing.

(b) Validation of CSB overexpression by Western blot with anti-flag and anti-CSB antibodies. β-actin served as a loading control.

(c) Validation of the IP efficiency of anti-flag antibody used in duplicated CSB ChIP-seq experiments. Input: fragmented chromatin before IP; FT: flow through chromatin fraction after IP; IP: flag enriched chromatin fraction.

(d) Correlation between biological replicates of CSB ChIP-seq libraries based on normalized reads per million (RPM).

(e) Pie plot showing the distribution of CSB ChIP-seq peaks in HEK293 cells.

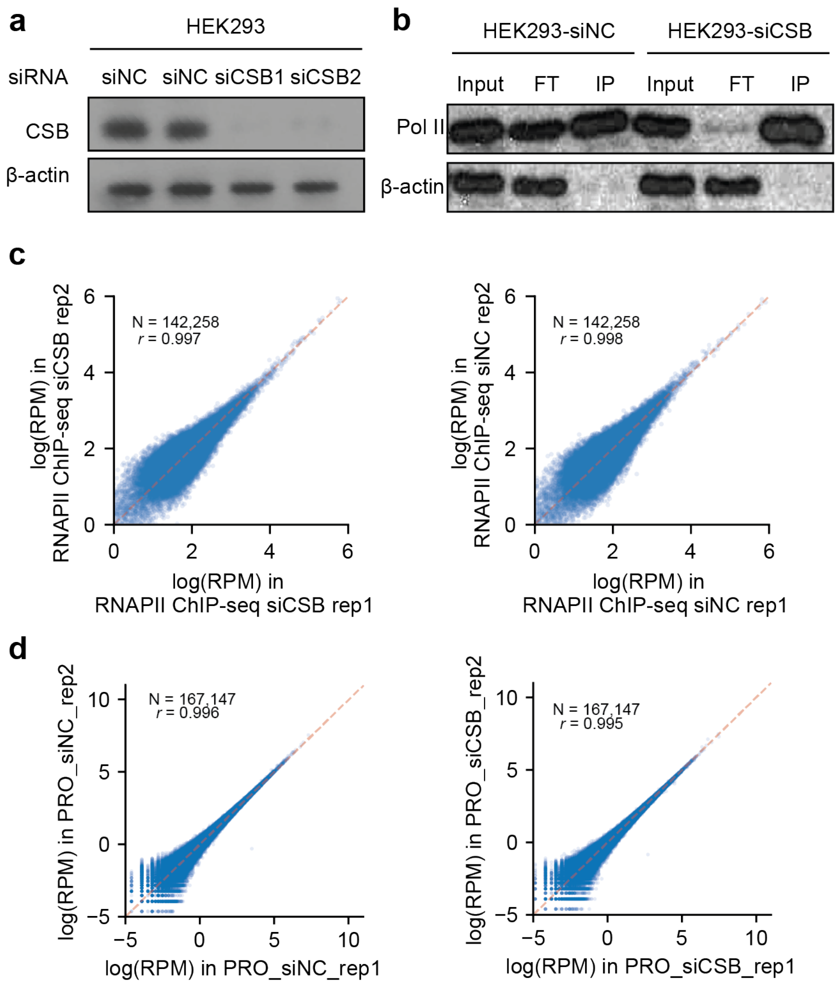

Supplementary Figure 6. Quality control for Pol II ChIP-seq and PRO-seq.

(a) Western blot validation of CSB knockdown efficiency in siNC and siCSB-treated cells after 72 hours in comparison with siNC-treated cells. NC: scrambled siRNA serving as a negative control.

(b) Western blot validation of the IP efficiency with anti-Pol II antibody (8WG16) used in Pol II ChIP-seq. Input: fragmented chromatin fraction before IP; FT: flow through chromatin fraction after IP; IP, Pol II antibody-enriched chromatin fraction.

(c) Correlation between biological replicates of Pol II ChIP-seq libraries generated from siNC and siCSB-treated HEK293 cells.

(d) Comparison between PRO-seq libraries generated from siNC and siCSB-treated HEK293 cells.

.

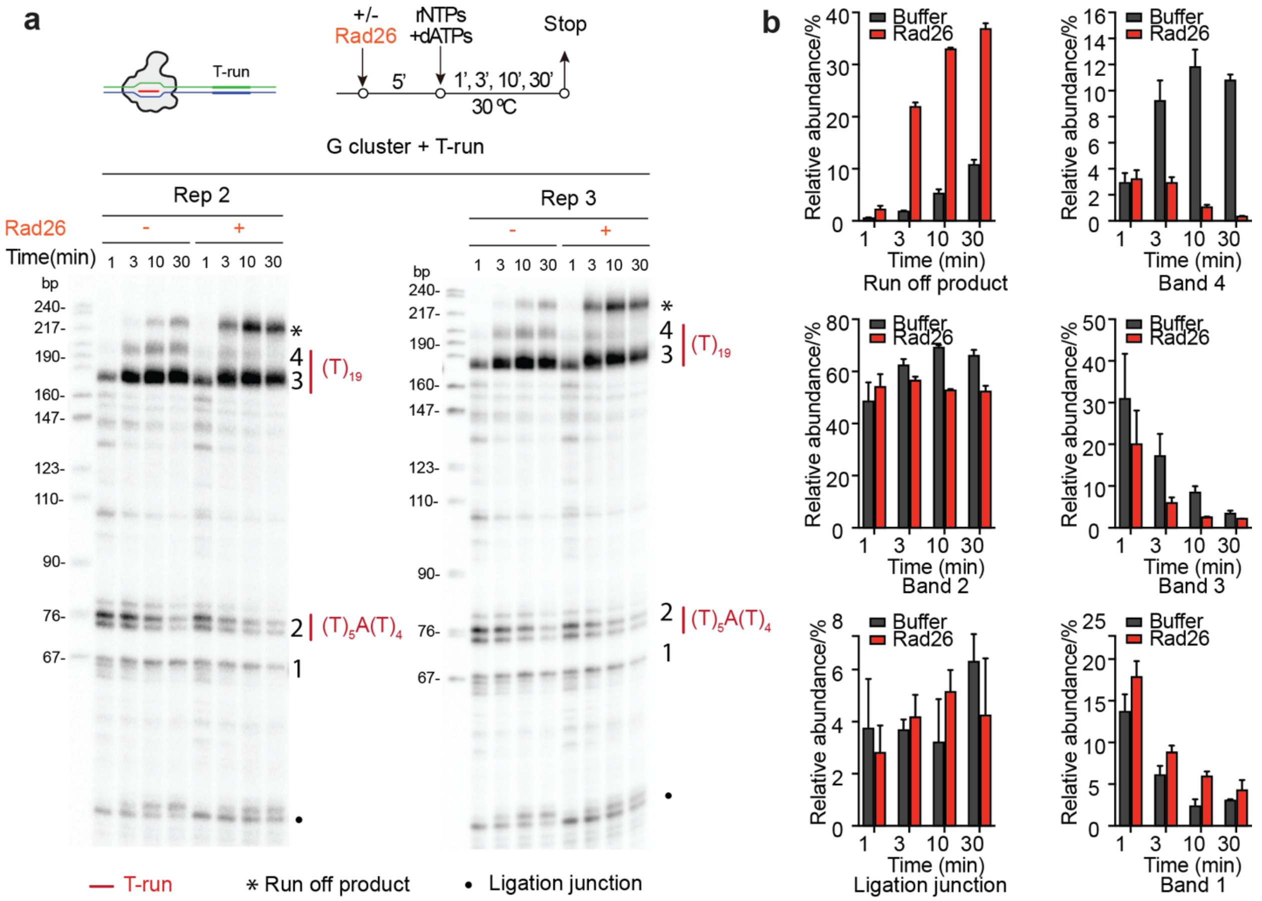

Supplementary Figure 7. CSB-dependent bypass of Pol II pausing at T-runs.

(a) Replicated assay of in vitro run-off in the absence or presence of the yeast CSB ortholog Rad26, showing its role in promoting Pol II bypass across the two indicated T-runs.

(b) Bar plots showing the quantitation of individual bands from the run-off assay. Data are presented as means ± SEM (n=3 biological replicates).

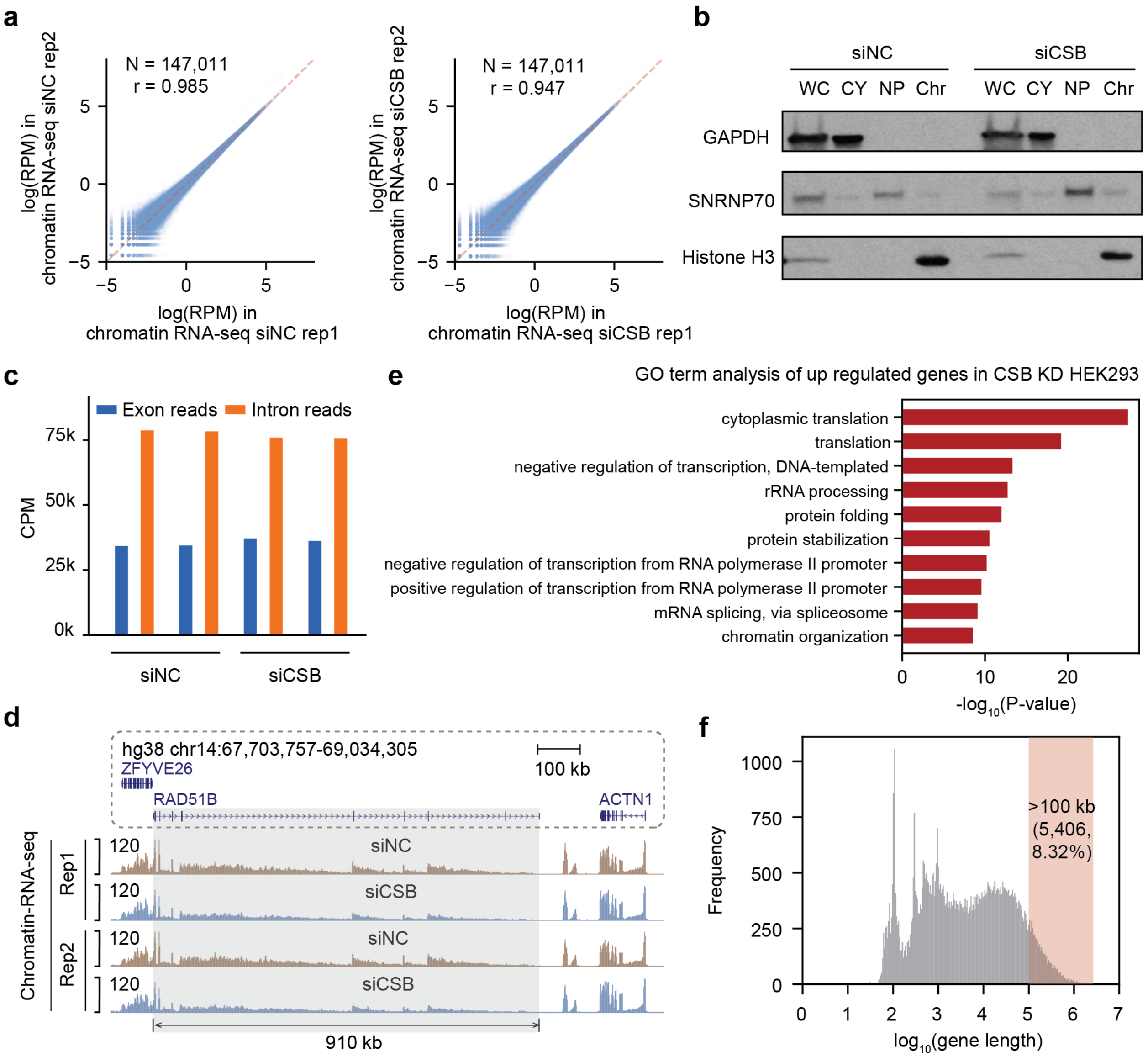

Supplementary Figure 8. Quality control for RNA-seq analysis and selective impact of CSB KD on relatively long genes.

(a) Correlation between biological replicates of chromatin associated RNA-seq libraries.

(b) Western blot validation of marker proteins in subcellular fractionation used in chromatin associated RNA-seq. GAPDH, SNRNP70 and histone H3 are markers for the cytoplasmic, nucleoplasm and chromatin/particle fractions, respectively. WC: whole cell; CY: cytosolic fractions; NP: nucleoplasm; Chr: chromatin/particle fraction.

(c) Exon-reads and intron reads from normalized (based on CPM, counts per million) chromatin associated RNA-seq libraries.

(d) Genome browser view, showing changes in RNA-seq signals of the long gene RAD51B in comparison with two two surrounding two short genes under siNC and siCSB conditions. Gene annotations are shown on top of the tracks.

(e) GO term analysis of tup-regulated genes identified by chromatin associated RNA-seq in normal versus CSB KD HEK293 cells.

(f) Gene length distribution across the human genome according to hg38 annotation.

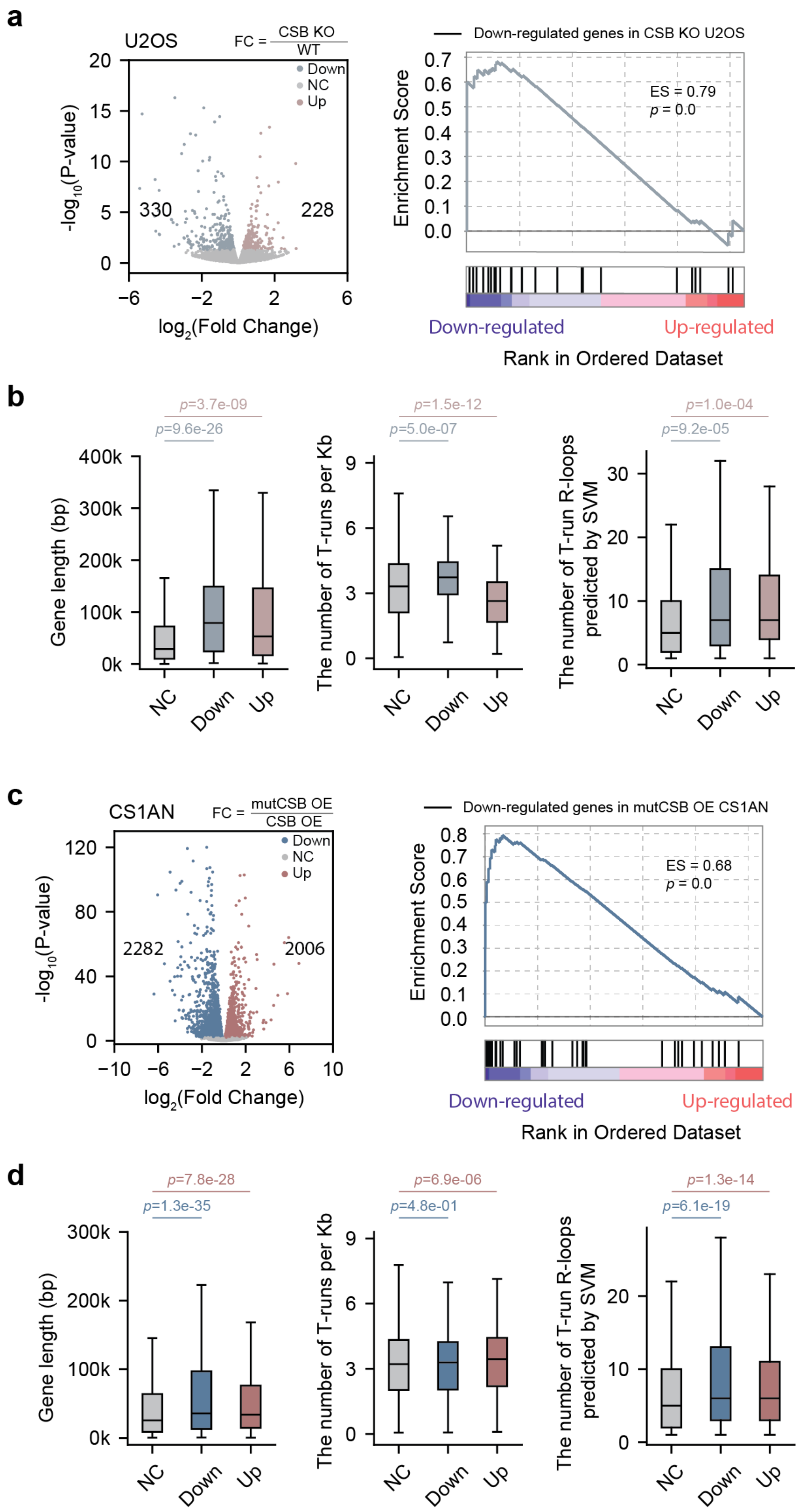

a
U2OS
FC = CSB KO / WT
Down
NC
Up
330
228
-log10(P-value)
log2(Fold Change)
Down-regulated genes in CSB KO U2OS
ES = 0.79
p = 0.0
Enrichment Score
Down-regulated
Up-regulated
Rank in Ordered Dataset
b
p=3.7e-09
p=9.6e-26
Gene length (bp)
p=1.5e-12
p=5.0e-07
The number of T-runs per Kb
p=1.0e-04
p=9.2e-05
The number of T-run R-loops predicted by SVM
NC
Down
Up
c
CS1AN
FC = mutCSB OE / CSB OE
Down
NC
Up
2282
2006
-log10(P-value)
log2(Fold Change)
Down-regulated genes in mutCSB OE CS1AN
ES = 0.68
p = 0.0
Enrichment Score
Down-regulated
Up-regulated
Rank in Ordered Dataset
d
p=7.8e-28
p=1.3e-35
Gene length (bp)
p=6.9e-06
p=4.8e-01
The number of T-runs per Kb
p=1.3e-14
p=6.1e-19
The number of T-run R-loops predicted by SVM
NC
Down
Up

Supplementary Figure 9. Enrichment of T-run-associated R-loops in genes regulated by CSB.

(a) Differential gene expression analysis on wildtype (WT) and CSB knockout (KO) U2OS cells. Left: Volcano plot of differentially expressed genes in response to CSB KD. Right: Down-regulated genes in CSB ko U2OS cells relative to those in CSB KD HEK293 cells.

(b) The distribution of gene lengths (left), T-runs per kb in each gene (middle), and SVM-predicted T-run associated R-loops (right) in wt versus CSB ko U2OS cells.

(c) Differential gene expression analysis on CSB deficient (mutCSB) vs CSB over expressing (CSB OE) CS1AN cells. Left: Volcano plot of differentially expressed genes in response to CSB KD. Right: Down-regulated genes in CSB deficient CS1AN cells relative to those in CSB KD HEK293 cells.

(d) The distribution of gene lengths (left), T-runs per kb in each gene (middle), and SVM-predicted T-run associated R-loops (right) in CSB deficient versus CSB expressing CS1AN cells.

For the box plots, the center line represents the median, while the upper and lower edges indicate the interquartile range. The whiskers extend to 1.5 times the interquartile range. Statistical significance was assessed using a two-tailed Mann–Whitney U-test, with sample sizes of 15240, 330, and 228 for unchanged, downregulated, and upregulated genes in plots (b), and 10322, 2282, and 2006 for not significant, downregulated, and upregulated genes in plots (d).

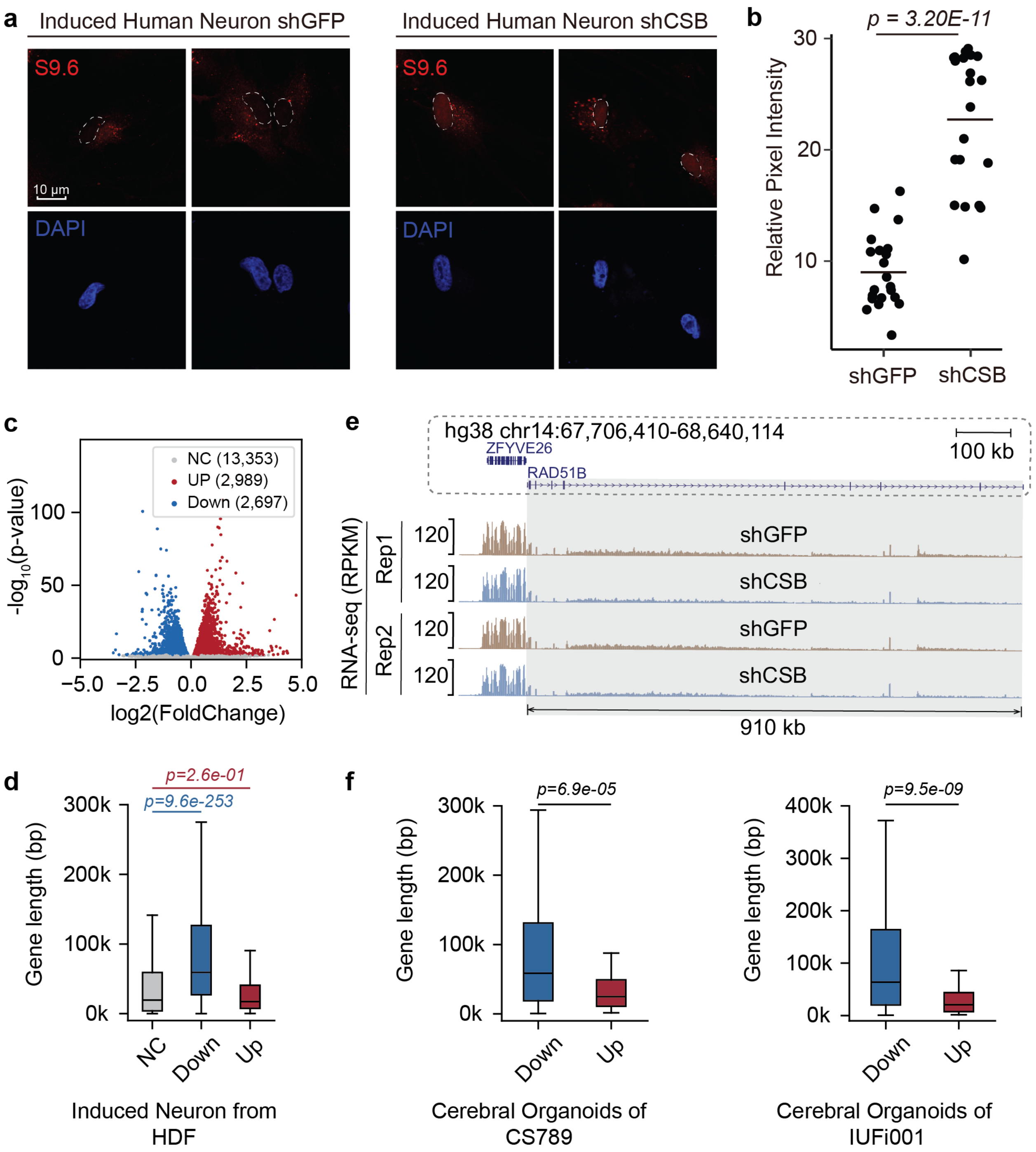

Supplementary Figure 10. Impact of CSB deficiency on long genes in PTBP1 depleted Human Dermal Fibroblasts (HDFs) and cerebral organoids.

(a) Immunofluorescence images displaying S9.6 signals (R-loop marker, stained in red) and nuclei (stained in blue) in differentiated HDFs induced by PTBP1 KD. Note: Cytosolic S9.6 signals may represent various RNA structures cross-reacted with the antibody.

(b) Quantitative analysis of S9.6 staining intensity in PTBP1 KD HDFs. Statistical analyses were conducted using a two-tailed unpaired t-test (n=20). The p-values are indicated above the respective graphs.

(c) Volcano plot displaying differentially expressed genes before and after CSB KD in HDFs.

(d) Gene length distribution within unchanged, downregulated, and upregulated gene sets.

(e) Genome browser tracks demonstrating changes in RNA-seq signals on a relative long gene RAD51B in comparison with a short multiple-exon gene ZFYVE26 in HDFs treated with shGFP versus shCSB. Gene annotations are indicated above the tracks.

(f) Gene length distribution within downregulated and upregulated gene sets resulting from the analysis of differential gene expression in RNA-seq data comparing healthy control cerebral organoids with cerebral organoids derived from two Cockayne Syndrome patients, CS789 and IUFi001.

For the box plots, the center line represents the median, while the upper and lower edges indicate the interquartile range. The whiskers extend to 1.5 times the interquartile range. Statistical significance was assessed using a two-tailed Mann–Whitney U-test, with sample sizes of 13353, 2697, and 2989 for unchanged, downregulated, and upregulated genes in plots (b), and 259 and 49 for downregulated and upregulated genes in plots (f, left), and 240 and 69 for downregulated and upregulated genes in plots (f, right).

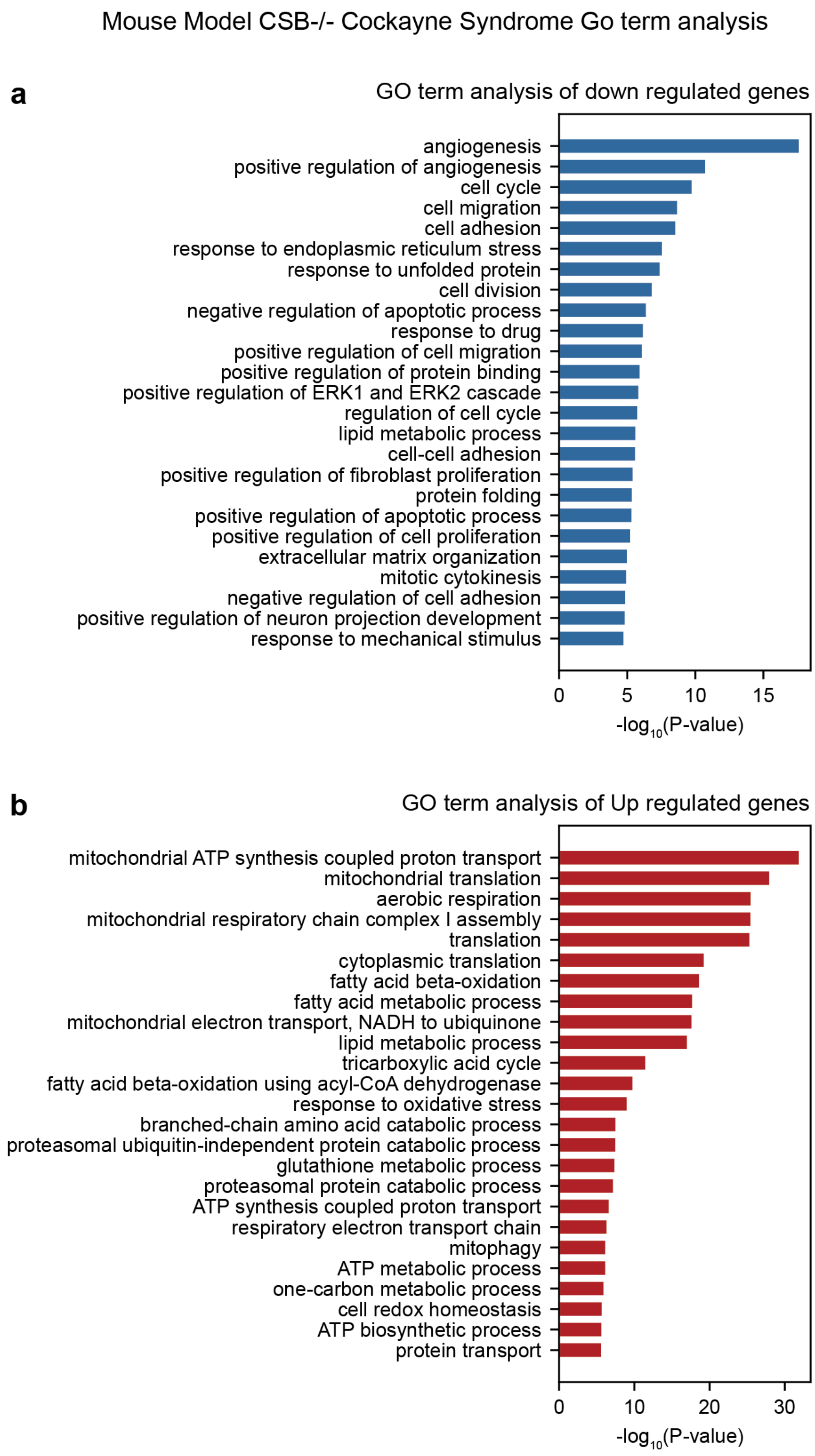

Supplementary Figure 11. GO term analysis on the mouse model of Cockayne Syndrome.

(a and b) Go term analysis of downregulated (a) and upregulated (b) genes in CSB-/- mice.

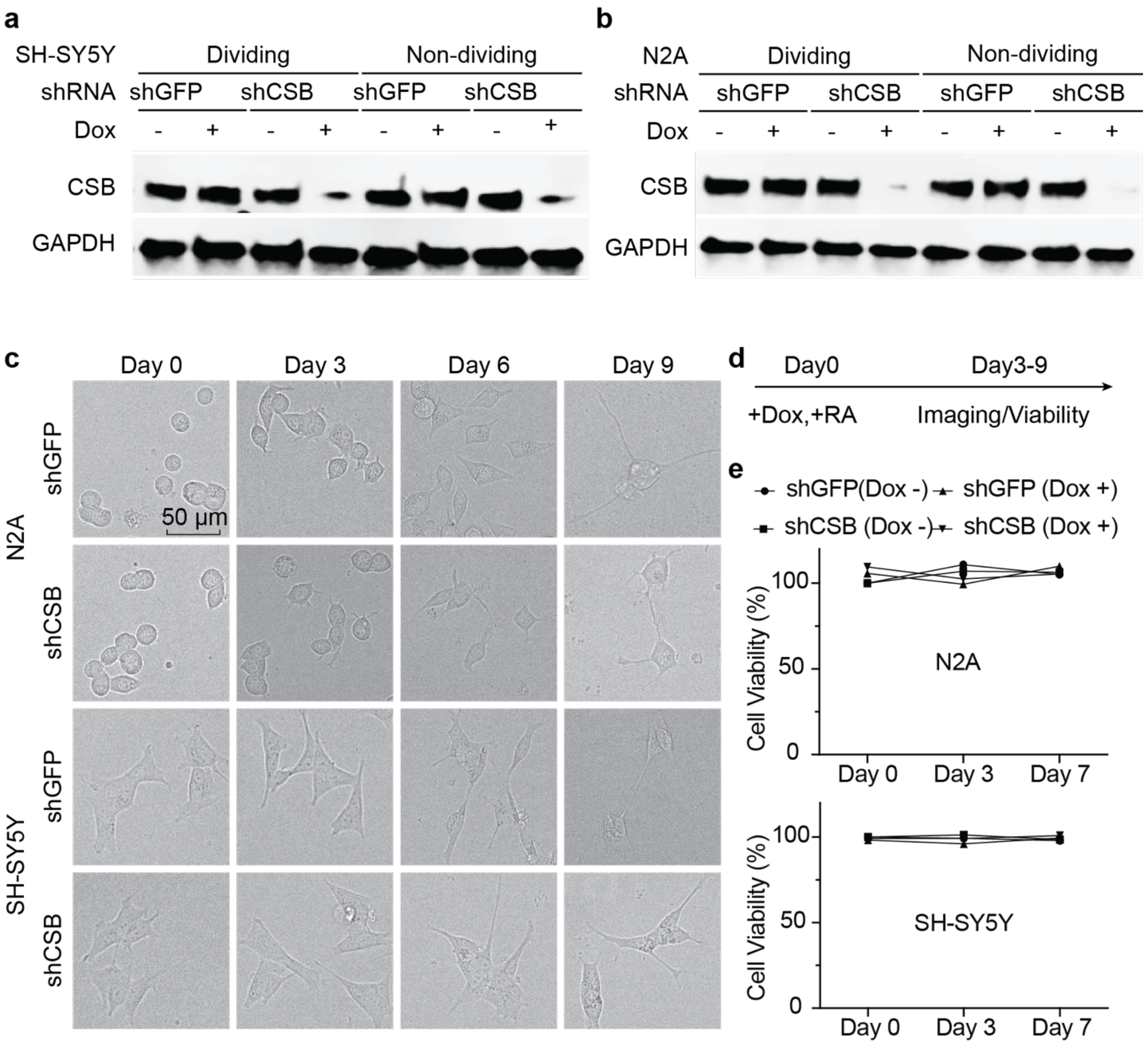

Supplementary Figure 12. Effect of CSB KD on neuronal differentiation and viability in SH-SY5Y and N2A Cells.

(a and b) Western blot analysis illustrating the impact of doxycycline-induced CSB KD in human SH-SY5Y (a) and mouse N2A (b) cells during the proliferative phase and after 14 days of differentiation into non-dividing cells.

(c and d) Microscopic images illustrating the morphological changes (c) observed during the differentiation of N2A and SH-SY5Y cells induced by retinoic acid (RA) at various time points (d). Cells were either treated with shGFP (control) or shCSB (CSB KD) before RA-induced differentiation.

(e) Cell viability assessment depicting changes throughout the differentiation process in mouse N2A (top) and human SH-SY5Y (bottom) cells.

Table S1: RNA and DNA oligos used in this study

**siRNA oligos for knockdown**		
Target Gene	Strand	Sequence 5’-->3’
ERCC6_1	Sense	CAGAACACGAUGACCUUCUdTdT
	Antisense	AGAAGGUCAUCGUGUUCUGdTdT
ERCC6_2	Sense	CAGGAAACUAGAUUCUGUAdTdT
	Antisense	UACAGAAUCUAGUUUCCUGdTdT
Negative Control	Sense	UUCUCCGAACGUGUCACGUdTdT
	Antisense	ACGUGACACGUUCGGAGAAdTdT

**DNA oligos for R-ChIP library construction**	
Primer Name	Sequence
N9 random primer (Illumina HiSeq 2500 system)	5′-/Invddt/CAAGCAGAAGACGGCATACGAGNNNNNNNNN-3′
Oligo A	5′-/Phos/GATCGGAAGAGCGTCGTGTAGGGAAAGAGTGT-3′
Oligo B	5′-AGACGTGTGCTCTTCCGATCT-3′
PCR primer	5′-CAAGCAGAAGACGGCATACGAG-3′
Barcode primer	5′-AATGATACGGCGACCACCGAGATCTACACNNNNNACACTCTTTCCCTACACGACGCTCTTCCGATCT-3′

**Primers for *in vitro* transcription**	
Primer Name	Sequence
T_Rloop_pair1_rev	5′-CAAAAAAAAAAAAAAAAAAAGAATGTTTACCAATAGTGACTCTCTGAAACCCCTCACCAC-3′
T_Rloop_pair1_fwd	5′-AGAAAAGTAGTGTACGAGTTAACATAGAAGTGGTTCAGGGTGGGAGTGGTGAGGGGTTTC-3′
T_Rloop_pair2_rev	5′-TGCGCCATTGCACTCCAGCCTGGGCAATAGAGCAAGACTCTGTCTCAAAAAAAAAAAAAA-3′
T_Rloop_pair2_fwd	5′-GAGACACCGGCACTGGGAGCCGAGTAGGTGGAATTTTTATTTTGCAGAAAAGTAGTGTAC-3′
Control	5-GAGGAGTAGAGAAGGAGATGGAGCTCGGTACCCGGGGATCCTCTAGAGTGGGAGCTCGG AACACTATCCGACTGGCACCGGCAAGGTCGCTGTTCAATACATGCACAGGATGTATATAT CTGACACGTGCCTGGAGACTAGGGAGAGAGGAGAGAGAGAAGAGAAGAGAGAGGCATGTCGG-3'
RNA10	5’-AUCGAGAGGA-3'
TS	5’-GGTGTCTCTTGGGTTGGCTCTTCGCCTTCTGTCCTCTC GATATCTGAATTCGTATAGGGTCCATCACATAAGGGATG-3'
NTS	5’-Biotin-TGTCATCCCTTATGTGATGGACCCTATACGAATTCAGATATCG AGAGGACAGAAGGCGAAGAGCCAACCCAAGAGACACCGGCACTGGG-3'

**Table S2: Global run-on reaction mix**		
Reagent	Volume	Final concentration
Run on buffer	780ul	NA
10% sarkosyl (final 1%)	100ul	1%
100mM ATP	5ul	500 uM
100mM GTP	5ul	500 uM
1mM Biotin-UTP	50ul	50uM
1mM Biotin-CTP	50ul	50uM
superase	10ul	NA
0.1M DTT	10ul	1mM
Total	1010ul	
