# Supplementary material for: Cockayne Syndrome Linked to Elevated R-Loops Induced by Stalled RNA Polymerase II during Transcription Elongation
Source: Nat Commun. 2024 Jul 17;15:6031. doi: 10.1038/s41467-024-50298-w (PMC11255242; doi:10.1038/s41467-024-50298-w)
Supplement: Supplementary file 3 — Reporting Summary [file 41467_2024_50298_MOESM3_ESM.pdf]

nature portfolio

Corresponding author(s): Xiang-Dong Fu
Dong Wang

Last updated by author(s): May 26, 2024

Reporting Summary

Nature Portfolio wishes to improve the reproducibility of the work that we publish. This form provides structure for consistency and transparency in reporting. For further information on Nature Portfolio policies, see our Editorial Policies and the Editorial Policy Checklist.

Statistics

For all statistical analyses, confirm that the following items are present in the figure legend, table legend, main text, or Methods section.

n/a	Confirmed	
☐	☒	The exact sample size (*n*) for each experimental group/condition, given as a discrete number and unit of measurement
☐	☒	A statement on whether measurements were taken from distinct samples or whether the same sample was measured repeatedly
☐	☒	The statistical test(s) used AND whether they are one- or two-sided<br>*Only common tests should be described solely by name; describe more complex techniques in the Methods section.*
☒	☐	A description of all covariates tested
☒	☐	A description of any assumptions or corrections, such as tests of normality and adjustment for multiple comparisons
☐	☒	A full description of the statistical parameters including central tendency (e.g. means) or other basic estimates (e.g. regression coefficient) AND variation (e.g. standard deviation) or associated estimates of uncertainty (e.g. confidence intervals)
☐	☒	For null hypothesis testing, the test statistic (e.g. *F*, *t*, *r*) with confidence intervals, effect sizes, degrees of freedom and *P* value noted<br>*Give P values as exact values whenever suitable.*
☒	☐	For Bayesian analysis, information on the choice of priors and Markov chain Monte Carlo settings
☒	☐	For hierarchical and complex designs, identification of the appropriate level for tests and full reporting of outcomes
☐	☒	Estimates of effect sizes (e.g. Cohen's *d*, Pearson's *r*), indicating how they were calculated

Our web collection on statistics for biologists contains articles on many of the points above.

Software and code

Policy information about availability of computer code

Data collection	Illumina Nova seq 4000
Data analysis	Details of how to use these software is described in the method section, such as BWA 0.7.17-r1188, STAR 2.7.1a, Samtools 1.6, MACS 2.2.7.1, Bedtools v2.30.0, trim_galore 0.6.7, featureCounts 1.6.3, homer v 4.11.1, MEME 5.4.1, deepTools 3.5.1, Deseq2 1.38.3, GSEA Linux 4.2.3, Picard 2.20.3-SNAPSHOT

For manuscripts utilizing custom algorithms or software that are central to the research but not yet described in published literature, software must be made available to editors and reviewers. We strongly encourage code deposition in a community repository (e.g. GitHub). See the Nature Portfolio guidelines for submitting code & software for further information.

Data

Policy information about availability of data

All manuscripts must include a data availability statement. This statement should provide the following information, where applicable:

- Accession codes, unique identifiers, or web links for publicly available datasets
- A description of any restrictions on data availability
- For clinical datasets or third party data, please ensure that the statement adheres to our policy

The raw data generated or analyzed during this study have been deposited into NCBI GEO under the accession number GEO: GSE226204.
Additionally, all source data are available at https://doi.org/10.6084/m9.figshare.22207912

Research involving human participants, their data, or biological material

Policy information about studies with human participants or human data. See also policy information about sex, gender (identity/presentation), and sexual orientation and race, ethnicity and racism.

Reporting on sex and gender	NA
Reporting on race, ethnicity, or other socially relevant groupings	NA
Population characteristics	NA
Recruitment	NA
Ethics oversight	NA

Note that full information on the approval of the study protocol must also be provided in the manuscript.

Field-specific reporting

Please select the one below that is the best fit for your research. If you are not sure, read the appropriate sections before making your selection.

☒ Life sciences ☐ Behavioural & social sciences ☐ Ecological, evolutionary & environmental sciences

For a reference copy of the document with all sections, see nature.com/documents/nr-reporting-summary-flat.pdf

Life sciences study design

All studies must disclose on these points even when the disclosure is negative.

Sample size	No statistical analysis was employed to determine the sample size. The chosen sample size was determined based on prior publications.
Data exclusions	None
Replication	All experiments in the manuscript have been repeated. All statistical analysis were based on at least three replicates.
Randomization	NA
Blinding	NA

Reporting for specific materials, systems and methods

We require information from authors about some types of materials, experimental systems and methods used in many studies. Here, indicate whether each material, system or method listed is relevant to your study. If you are not sure if a list item applies to your research, read the appropriate section before selecting a response.

Materials & experimental systems

n/a	Involved in the study	
☐	☒	Antibodies
☐	☒	Eukaryotic cell lines
☒	☐	Palaeontology and archaeology
☒	☐	Animals and other organisms
☒	☐	Clinical data
☒	☐	Dual use research of concern
☒	☐	Plants

Methods

n/a	Involved in the study	
☐	☒	ChIP-seq
☒	☐	Flow cytometry
☒	☐	MRI-based neuroimaging

Antibodies

Antibodies used	Mouse anti-V5 tag Invitrogen Cat# R96025; RRID: AB_2556564<br>Mouse anti-FLAG tag Sigma-Aldrich Cat# F1804; RRID: AB_262044<br>Rabbit anti-CSB Bethyl Cat# A301-345A; RRID: AB_937849<br>Rabbit anti-GAPDH Cell Signaling Technology Cat# 5014; RRID: AB_10693448

	Mouse anti-β-actin Sigma-Aldrich Cat# A5441; RRID: AB_476744<br>Mouse anti-SNRNP70 Santa Cruz Biotechnology Cat# sc-390899; RRID: AB_2801569<br>Mouse anti-RPB1 (8WG16) Santa Cruz Biotechnology Cat# sc-56767; RRID: AB_785522<br>Rabbit anti-Histone H3 Cell Signaling Technology Cat# 9715; RRID: AB_331563<br>Goat anti-Rabbit HRP-linked IgG Cell Signaling Technology Cat# 7074; RRID: AB_2099233<br>Horse anti-Mouse HRP-linked IgG Cell Signaling Technology Cat# 7076; RRID: AB_330924
Validation	The antibodies used were validated by the previous publications or routine experiment protocol, listed in the method section.

Eukaryotic cell lines

Policy information about cell lines and Sex and Gender in Research

Cell line source(s)	HEK293, HEK293T, N2A and SH-SY5Y cells were from a common laboratory stock. Primary human dermal fibroblasts were purchased from ATCC PCS-201-012.
Authentication	The cell lines used were checked for morphology by microscope.
Mycoplasma contamination	We periodically checked the potential contamination with mycoplasma using PCR based mycoplasma detection kit.
Commonly misidentified lines<br>(See ICLAC register)	None.

Plants

Seed stocks	NA
Novel plant genotypes	NA
Authentication	NA

ChIP-seq

Data deposition

☒ Confirm that both raw and final processed data have been deposited in a public database such as GEO.

☒ Confirm that you have deposited or provided access to graph files (e.g. BED files) for the called peaks.

Data access links<br>*May remain private before publication.*	https://www.ncbi.nlm.nih.gov/geo/query/acc.cgi?acc=GSE226204
Files in database submission	ChIP-seq, PRO-seq, RNA-seq
Genome browser session<br>(e.g. UCSC)	NA

Methodology

Replicates	Each experiment has two replicates.
Sequencing depth	R-ChIP used the single-end sequencing, the rest of the data used the pair-end sequencing. The read depth is around 20 M, the uniq mapping rate is around 96% on average. Length of reads was reflected in our GEO database.
Antibodies	Mouse anti-V5 tag Invitrogen Cat# R96025; RRID: AB_2556564<br>Mouse anti-FLAG tag Sigma-Aldrich Cat# F1804; RRID: AB_262044
Peak calling parameters	All alignment detailed include the options used in the command line is in the method part of our manuscript.
Data quality	QValue larger than 10 is the threshold we used to callpeaks using the macs2 software. For the details, refer to the method part.
Software	BWA 0.7.17-r1188, STAR 2.7.1a, Samtools 1.6, MACS 2.2.7.1, Bedtools v2.30.0, trim_galore 0.6.7, featureCounts 1.6.3
